# Synchrotron study of the garnet-type oxide Li_6_CaSm_2_Ta_2_O_12_

**DOI:** 10.1107/S1600536809040008

**Published:** 2009-10-07

**Authors:** Chung-Yul Yoo, Sung-Chul Kim, Seung-Soo Lee, Seung-Joo Kim

**Affiliations:** a Department of Chemistry, Division of Energy Systems Research, Ajou University, Suwon 443-749, Republic of Korea; b Institute of NT-IT Fusion Technology, Ajou University, Republic of Korea

## Abstract

Hexalithium calcium disamarium(III) ditantalum(V) dodeca­oxide, Li_6_CaSm_2_Ta_2_O_12_, crystallizes in a cubic garnet-type structure. In the crystal structure, disordered Li atoms occupy two crystallographic sites. One Li has a tetra­hedral coordination environment in the oxide lattice, whereas the other Li atom occupies a significantly distorted octa­hedral site, with site occupancies for the two Li atoms of 0.26 (7) and 0.44 (2), respectively. Ca and Sm atoms are statistically distributed over the same crystallographic position with a occupancy of 2/3 for Sm and of 1/3 for Ca, and are eightfold coordinated by O atoms. The TaO_6_ octa­hedron is joined to six others *via* corner-sharing LiO_4_ tetra­hedra. One Li and the O atoms lie on general positions, while the other atoms are situated on special positions. The Sm/Ca position has 222, Ta has 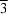, and the tetra­hedrally coordinated Li atom has 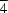 site symmetry.

## Related literature

For a general description of structures and physical properties of garnets, see: Geller (1967 ▶). Recently, high Li-ion conductivity was discovered in garnet-related compounds such as Li_5_La_3_ *M*_2_O_12_ (*M* = Nb, Ta), see: Thangadurai *et al.* (2003 ▶); Cussen (2006 ▶). For studies focused on the substitution of La^3+^ by divalent alkaline earth ions (Ca, Sr, Ba), see: Murugan *et al.* (2007 ▶); Thangadurai & Weppner (2005*a* ▶,*b* ▶); O’Callaghan & Cussen (2007 ▶); Percival & Slater (2007 ▶). For indexing the powder diffraction pattern, see: Boultif & Louër (2004 ▶).

## Experimental

### 

#### Crystal data

Li_6_CaSm_2_Ta_2_O_12_
*M* *_r_* = 936.45
Cubic, 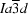

*a* = 12.55128 (7) Å
*V* = 1977.26 (2) Å^3^
*Z* = 8
Synchrotron radiation
λ = 1.54900 Å
*T* = 298 K
Specimen shape: flat sheet
20 × 20 × 0.5 mm
Specimen prepared at 103 kPa
Specimen prepared at 1223 K
Particle morphology: particle, yellowish-white

#### Data collection

Pohang Light Source 8C2 HRPD Beamline diffractometer
Specimen mounting: ’packed powder pellet’
Specimen mounted in reflection mode
Scan method: step
2θ_min_ = 10.0, 2θ_max_ = 131.0°
Increment in 2θ = 0.01°

#### Refinement

*R*_p_ = 15.0
*R*_wp_ = 22.0
*R*_exp_ = 13.1
*R*_B_ = 6.62
*S* = 1.67
Excluded region(s): None
Profile function: pseudo Voigt
151 reflections
20 parameters
Preferred orientation correction: none

### 

Data collection: local software at 8C2 HRPD beamline; cell refinement: *FULLPROF* (Rodriguez-Carvajal, 2001 ▶); data reduction: *FULLPROF*; program(s) used to solve structure: *SHELXS97* (Sheldrick, 2008 ▶); program(s) used to refine structure: *FULLPROF*; molecular graphics: *DIAMOND* (Brandenburg, 1999 ▶); software used to prepare material for publication: *FULLPROF*.

## Supplementary Material

Crystal structure: contains datablocks global, I. DOI: 10.1107/S1600536809040008/wm2261sup1.cif

Rietveld powder data: contains datablocks I. DOI: 10.1107/S1600536809040008/wm2261Isup2.rtv

Additional supplementary materials:  crystallographic information; 3D view; checkCIF report

## Figures and Tables

**Table 1:** Selected bond lengths (Å)

(Sm,Ca)—O1	2.561 (17)
Ta1—O1	2.014 (6)
Li1—O1	1.843 (7)
Li2—O1^i^	1.63 (6)
Li2—O1^ii^	2.14 (6)
Li2—O1^iii^	2.12 (6)
Li2—O1^iv^	2.20 (6)
Li2—O1^v^	2.55 (6)
Li2—O1^vi^	2.69 (6)

Symmetry codes: (i) 
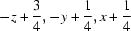
; (ii) 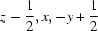; (iii) 
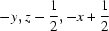
; (iv) 
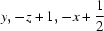
; (v) 
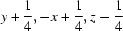
; (vi) 
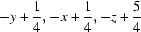
.

**Fig. 1.**
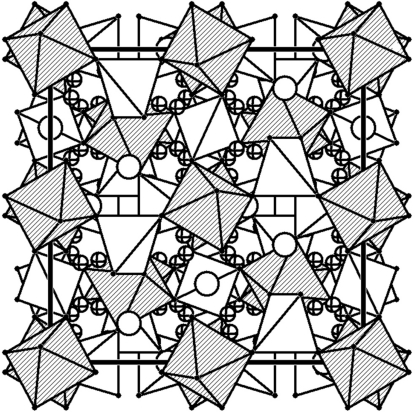
Schematic representation of the crystal structure of Li6CaSm2Ta2O12; the crossed spheres and the hollow spheres represent Li and Sm/Ca atoms, respectively.

**Fig. 2.**
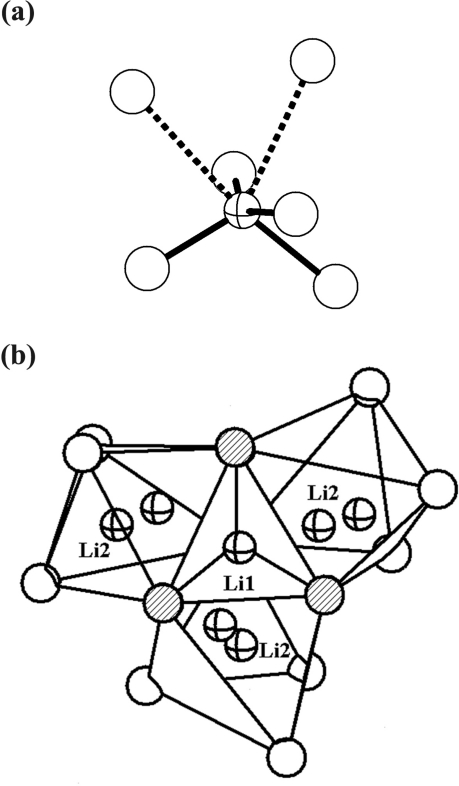
(a) Local environment of Li2 site: The crossed spheres and the hollow spheres represent lithium and oxygen atoms, respectively. The relatively long Li—O bonds are dotted. (b) The Li1O4 tetrahedron shares each face with a Li2O6 octahedron.

**Fig. 3.**
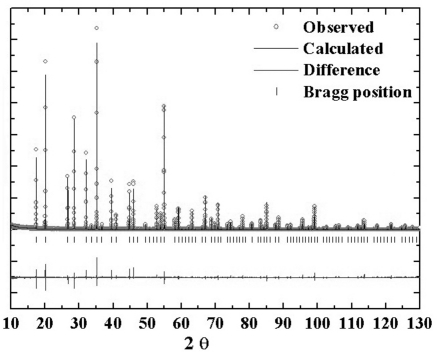
Rietveld refinement plot of Li6CaSm2Ta2O12 based on synchrotron X-ray powder diffraction data.

## supplementary crystallographic information

### Comment

Conventional garnet-type oxides with general formula *A*_3_*B*_3_*C*_2_O_12_ contain tetrahedral, cubic and octahedral coordination environments filled with *A*, *B*, and *C* atoms, respectively. The garnet structure has attracted renewed interest since a Li^+^ ionic conductivity was observed in the compound Li_5_La_3_Ta_2_O_12_, which contains an excess of Li beyond the usual garnet composition. The ionic conductivity was enhanced through the increase of the Li content *via* partial substitution of trivalent La^3+^ by divalent alkaline earth ions (Murugan *et al.*, 2007). The structure of the title compound is closely related to that of Li_6_SrLa_2_Ta_2_O_12_ (Percival & Slater, 2007). Li1 atoms are located at site 24*d* (tetrahedral), Li2 atoms at site 96*h* (distorted octahedral), Sm/Ca atoms are at site 24*c* (cubic), Ta atoms at site 16*a* (octahedral), and O atoms at general site 96*h* (Fig. 1). As shown in Fig. 2, the partially occupied Li2 site exhibits a significantly distorted [4 + 2] coordination polyhedron with Li—O bond lengths between 1.63 (6) - 2.69 (6) Å. The Li1 atoms at the tetrahedral sites and adjacent Li2 atoms at the octahedral sites are connected by common oxygen atoms *via* face-sharing. Considering the site occupation factors (SOF) for Li1 and Li2 sites, Li_6_CaSm_2_Ta_2_O_12_ can be described as Li2_(3+_*_x_*_)_[Li1(_3-_*_x_*_)_(Ca_1/3_Sm_2/3_)_3_Ta_2_O_12_] with *x* = 2.23.

For a general description of structures and physical properties of garnets, see: Geller (1967). High Li-ion conductivity was discovered in garnet-related compounds such as Li_5_La_3_*M*_2_O_12_, where *M* = Nb, Ta (Thangadurai *et al.*, 2003; Cussen, 2006). For studies focused on the substitution of La^3+^ by divalent alkaline earth ions (Ca, Sr, Ba), see: Thangadurai & Weppner (2005a,b), O'Callaghan & Cussen (2007).

### Experimental

The polycrystalline sample of Li_6_Sm_2_CaTa_2_O_12_ was prepared by solid-state reaction of stoichiometric amounts of Sm_2_O_3_, Ta_2_O_5_, CaCO_3_ and 10% excess of Li_2_CO_3_. Sm_2_O_3_ was preheated at 1173 K for 24 h to remove absorbed water before using. The finely ground samples were heated at 1123 and 1173 K for 12 h and then 1223 K for 24 h, with intermediate regrindings. Synchrotron X-ray diffraction (sXRD) measurement was performed on beamline 8 C2-HRPD at Pohang Accelerator Laboratory, Pohang, Korea. The incident X-rays were vertically collimated by a mirror, and monochromated to the wavelength of 1.5490 Å by double-crystal Si (111) monochromator. The datasets were collected in the range of 10° ≤ 2θ≤ 130° with a step size of 0.01° (2θ range).

### Refinement

All reflections could be indexed with a body centered cubic cell. Any additional peaks due to symmetry lowering or impurity phase were not detected. The unit-cell parameter was determined with the DICVOL program (Boultif & Louër, 2004). The figures of merit were *M*(20) = 175.8, F(20) = 134.8(0.0013, 114). The reflection conditions for (*hkl*): *h *+ *k *+ *l* = *even*, (*0kl*): *k*,*l* = *even*, (*hhl*): *2 h + l *= *4n* and *l* = *even*, (*00 l*): *l* = *4n* suggested that the Li containing-garnet structure belonged to *Ia*3*d* space group. The atomic positions of Ta, Sm, Ca and O atoms were determined employing direct methods using the synchrotron XRD data. The total amplitude factors (148, 'F_obs_') were converted into structure factors and used as an input for the *SHELXS97* program (Sheldrick, 2008). The positions of Li1 and Li2 were then determined by difference Fourier analysis in *SHELXL97* program (Sheldrick, 2008). Structure refinements of atomic positions, occupancy and isotropic displacement parameters were carried out by the Rietveld method using the FULLPROF program with pseudo-Voigt peak shapes and manually selected backgrounds (Rodriguez-Carvajal, 2001). In the refinement, the SOFs of two Li sites were constrained in such a way that the total amount of Li atoms was constant to maintain the chemical composition. The isotropic displacement parameters for two Li atoms were set to the same refined value because there was a strong correlation between the isotropic displacement parameters and the SOFs for both Li1 and Li2 sites. The Rietveld refinement plot based on the synchrotron XRD data is shown in Fig. 3.

### Crystal data

**Table:**

Li_6_CaSm_2_Ta_2_O_12_	*D*_x_ = 6.292 Mg m^−^^3^
*M**_r_* = 936.45	Synchrotron radiation, λ = 1.5490 Å
Cubic, *I**a*3*d*	*T* = 298 K
Hall symbol: -I 4bd 2c 3	Particle morphology: particle
*a* = 12.55128 (7) Å	yellowish-white
*V* = 1977.26 (2) Å^3^	flat sheet, 20 × 20 mm
*Z* = 8	Specimen preparation: Prepared at 1223 K and 103 kPa, cooled at 5 K/min K min^−^^1^
*F*(000) = 3200	

### Data collection

**Table:**

Pohang Light Source 8C2 HRPD Beamline diffractometer	Data collection mode: reflection
Radiation source: Synchrotron	Scan method: step
Si 111	2θ_min_ = 10.00°, 2θ_max_ = 131.00°, 2θ_step_ = 0.01°
Specimen mounting: 'packed powder pellet'	

### Refinement

**Table:**

*R*_p_ = 15.0	Profile function: pseudo Voigt
*R*_wp_ = 22.0	20 parameters
*R*_exp_ = 13.1	0 restraints
*R*_Bragg_ = 6.62	(Δ/σ)_max_ < 0.001
χ^2^ = 2.789	Background function: manual background
12100 data points	Preferred orientation correction: 'None'
Excluded region(s): None	

### Fractional atomic coordinates and isotropic or equivalent isotropic displacement parameters (Å^2^)

**Table:**

	*x*	*y*	*z*	*U*_iso_*/*U*_eq_	Occ. (<1)
Sm1	0.00000	0.25000	0.62500	0.0089 (2)*	0.6666
Ca1	0.00000	0.25000	0.62500	0.0089 (2)*	0.3333
Ta1	0.00000	0.00000	0.50000	0.0071 (2)*	
Li1	0.12500	0.00000	0.75000	0.0278 (11)*	0.26 (7)
Li2	0.101 (5)	0.192 (5)	0.412 (5)	0.0278 (11)*	0.44 (2)
O1	0.0323 (5)	0.0521 (5)	0.6488 (6)	0.0079 (13)*	

### Geometric parameters (Å, °)

**Table:**

(Sm,Ca)—O1^i^	2.441 (18)	Li1—O1^xiv^	1.843 (7)
(Sm,Ca)—O1^ii^	2.441 (18)	Li2—O1^xv^	1.63 (6)
(Sm,Ca)—O1^iii^	2.441 (18)	Li2—O1^xi^	2.14 (6)
(Sm,Ca)—O1^iv^	2.441 (18)	Li2—O1^i^	2.12 (6)
(Sm,Ca)—O1^v^	2.561 (17)	Li2—O1^iii^	2.20 (6)
(Sm,Ca)—O1	2.561 (17)	Li2—O1^xvi^	2.55 (6)
(Sm,Ca)—O1^vi^	2.561 (17)	Li2—O1^vii^	2.69 (6)
(Sm,Ca)—O1^vii^	2.561 (17)	Li1—Li2^viii^	1.53 (6)
Ta1—O1^viii^	2.014 (6)	Li1—Li2^xvii^	1.53 (6)
Ta1—O1	2.014 (6)	Li1—Li2^xviii^	1.53 (6)
Ta1—O1^ix^	2.014 (6)	Li1—Li2^xix^	1.53 (6)
Ta1—O1^x^	2.014 (6)	Li1—Li2^xx^	2.33 (6)
Ta1—O1^xi^	2.014 (6)	Li1—Li2^ix^	2.33 (6)
Ta1—O1^i^	2.014 (6)	Li1—Li2^vii^	2.33 (6)
Li1—O1^xii^	1.843 (7)	Li1—Li2^xxi^	2.33 (6)
Li1—O1	1.843 (7)	Li2—Li2^xxii^	2.27 (9)
Li1—O1^xiii^	1.843 (7)	Li2—Li2^xxiii^	2.27 (9)
			
O1^i^—(Sm,Ca)—O1^ii^	158.8 (8)	O1—Ta1—O1^x^	180.000 (1)
O1^i^—(Sm,Ca)—O1^iii^	72.8 (2)	O1^ix^—Ta1—O1^x^	87.2 (3)
O1^ii^—(Sm,Ca)—O1^iii^	111.2 (2)	O1^viii^—Ta1—O1^xi^	180.0 (4)
O1^i^—(Sm,Ca)—O1^iv^	111.2 (2)	O1—Ta1—O1^xi^	92.8 (3)
O1^ii^—(Sm,Ca)—O1^iv^	72.8 (2)	O1^ix^—Ta1—O1^xi^	87.2 (3)
O1^iii^—(Sm,Ca)—O1^iv^	158.8 (8)	O1^x^—Ta1—O1^xi^	87.2 (3)
O1^i^—(Sm,Ca)—O1^v^	74.0 (2)	O1^viii^—Ta1—O1^i^	87.2 (3)
O1^ii^—(Sm,Ca)—O1^v^	124.5 (4)	O1—Ta1—O1^i^	87.2 (3)
O1^iii^—(Sm,Ca)—O1^v^	95.4 (2)	O1^ix^—Ta1—O1^i^	180.000 (2)
O1^iv^—(Sm,Ca)—O1^v^	67.2 (2)	O1^x^—Ta1—O1^i^	92.8 (3)
O1^i^—(Sm,Ca)—O1	67.9 (8)	O1^xi^—Ta1—O1^i^	92.8 (3)
O1^ii^—(Sm,Ca)—O1	95.4 (2)	O1^xii^—Li1—O1	113.7 (6)
O1^iii^—(Sm,Ca)—O1	124.5 (4)	O1^xii^—Li1—O1^xiii^	101.7 (3)
O1^iv^—(Sm,Ca)—O1	74.0 (2)	O1—Li1—O1^xiii^	113.7 (6)
O1^v^—(Sm,Ca)—O1	108.13 (16)	O1^xii^—Li1—O1^xiv^	113.7 (6)
O1^i^—(Sm,Ca)—O1^vi^	124.5 (4)	O1—Li1—O1^xiv^	101.7 (3)
O1^ii^—(Sm,Ca)—O1^vi^	74.0 (2)	O1^xiii^—Li1—O1^xiv^	113.7 (6)
O1^iii^—(Sm,Ca)—O1^vi^	67.2 (2)	O1^xv^—Li2—O1^i^	110 (3)
O1^iv^—(Sm,Ca)—O1^vi^	95.4 (2)	O1^xi^—Li2—O1^i^	87 (2)
O1^v^—(Sm,Ca)—O1^vi^	73.41 (16)	O1^xv^—Li2—O1^iii^	106 (3)
O1—(Sm,Ca)—O1^vi^	167.0 (2)	O1^xi^—Li2—O1^iii^	150 (10)
O1^i^—(Sm,Ca)—O1^vii^	95.4 (2)	O1^i^—Li2—O1^iii^	83 (2)
O1^ii^—(Sm,Ca)—O1^vii^	67.2 (2)	O1^xv^—Li2—O1^xvi^	87 (10)
O1^iii^—(Sm,Ca)—O1^vii^	74.0 (2)	O1^xi^—Li2—O1^xvi^	82 (2)
O1^iv^—(Sm,Ca)—O1^vii^	124.5 (4)	O1^i^—Li2—O1^xvi^	165 (10)
O1^v^—(Sm,Ca)—O1^vii^	167.0 (2)	O1^iii^—Li2—O1^xvi^	101 (2)
O1—(Sm,Ca)—O1^vii^	73.41 (16)	O1^xv^—Li2—O1^vii^	148 (10)
O1^vi^—(Sm,Ca)—O1^vii^	108.13 (16)	O1^xi^—Li2—O1^vii^	78.4 (19)
O1^viii^—Ta1—O1	87.2 (3)	O1^i^—Li2—O1^vii^	98 (10)
O1^viii^—Ta1—O1^ix^	92.8 (3)	O1^iii^—Li2—O1^vii^	74.8 (18)
O1—Ta1—O1^ix^	92.8 (3)	O1^xvi^—Li2—O1^vii^	67 (6)
O1^viii^—Ta1—O1^x^	92.8 (3)		

Symmetry codes: (i) −*y*, *z*−1/2, −*x*+1/2; (ii) −*z*+3/4, *y*+1/4, *x*+3/4; (iii) *y*, −*z*+1, −*x*+1/2; (iv) *z*−3/4, −*y*+1/4, *x*+3/4; (v) *y*−1/4, *x*+1/4, −*z*+5/4; (vi) −*x*, −*y*+1/2, *z*; (vii) −*y*+1/4, −*x*+1/4, −*z*+5/4; (viii) −*z*+1/2, −*x*, *y*+1/2; (ix) *y*, −*z*+1/2, *x*+1/2; (x) −*x*, −*y*, −*z*+1; (xi) *z*−1/2, *x*, −*y*+1/2; (xii) −*x*+1/4, −*z*+3/4, *y*+3/4; (xiii) −*x*+1/4, *z*−3/4, −*y*+3/4; (xiv) *x*, −*y*, −*z*+3/2; (xv) −*z*+3/4, −*y*+1/4, *x*+1/4; (xvi) *y*+1/4, −*x*+1/4, *z*−1/4; (xvii) *z*−1/4, *y*−1/4, *x*+3/4; (xviii) *z*−1/4, −*y*+1/4, −*x*+3/4; (xix) −*z*+1/2, *x*, −*y*+1; (xx) −*y*+1/4, *x*−1/4, *z*+1/4; (xxi) *y*, *z*−1/2, −*x*+1; (xxii) *y*−1/4, −*x*+1/4, −*z*+3/4; (xxiii) −*y*+1/4, *x*+1/4, −*z*+3/4.

## References

[bb1] Boultif, A. & Louër, D. (2004). *J. Appl. Cryst.* **37**, 724–731.

[bb2] Brandenburg, K. (1999). *DIAMOND.* Crystal Impact GbR, Bonn, Germany.

[bb3] Cussen, E. J. (2006). *Chem. Commun.* pp. 412–413. 10.1039/b514640b 16493817

[bb4] Geller, S. (1967). *Z. Kristallogr.* **125**, 1–47.

[bb5] Murugan, R., Thangadurai, V. & Weppner, W. (2007). *Ionics*, **13**, 195–203. 10.1002/anie.200701144 17803180

[bb6] O’Callaghan, M. P. & Cussen, E. J. (2007). *Chem. Commun.* pp. 2048–2050. 10.1039/b700369b 17713074

[bb7] Percival, J. & Slater, P. R. (2007). *Solid State Commun.* **142**, 355–357.

[bb8] Rodriguez-Carvajal, J. (2001). *FULLPROF* Commission on Powder Diffraction (IUCr), Newsletter 26, pp. 12–19.

[bb9] Sheldrick, G. M. (2008). *Acta Cryst.* A**64**, 112–122. 10.1107/S0108767307043930 18156677

[bb10] Thangadurai, V., Kaack, H. & Weppner, W. (2003). *J. Am. Ceram. Soc.* **86**, 437–440.

[bb11] Thangadurai, V. & Weppner, W. (2005*a*). *Adv. Funct. Mater.* **15**, 107–112.

[bb12] Thangadurai, V. & Weppner, W. (2005*b*). *J. Am. Ceram. Soc.* **88**, 411–418.

